# Occupational exposure to polycyclic aromatic hydrocarbons and risk of prostate cancer

**DOI:** 10.1186/s12940-021-00751-w

**Published:** 2021-06-21

**Authors:** Christine Barul, Marie-Elise Parent

**Affiliations:** 1 Epidemiology and Biostatistics Unit, Centre Armand-Frappier Santé Biotechnologie, Institut national de la recherche scientifique, Université du Québec, 531 Boulevard des Prairies, Laval, Québec H7V 1B7 Canada; 2 Department of Social and Preventive Medicine, School of Public Health, Université de Montréal, Montréal, Québec Canada; 3 University of Montreal’s Hospital Research Centre, Montréal, Québec Canada

**Keywords:** Prostate, Polycyclic aromatic hydrocarbons, Tumor, Occupational health, Case–control study

## Abstract

**Background:**

Several industries entailing exposure to polycyclic aromatic hydrocarbons (PAHs) are known or suspected carcinogens. A handful of studies have assessed the role of PAHs exposure in prostate cancer risk, but none has examined tumor aggressiveness or the influence of screening practices and detection issues. We aimed to examine the association between lifetime occupational exposure to PAHs and prostate cancer risk**.**

**Methods:**

Detailed work histories were collected from 1,929 prostate cancer cases (436 aggressive) and 1,994 controls from Montreal, Canada (2005–2012). Industrial hygienists applied the hybrid expert approach to assign intensity, frequency and certainty of exposure to benzo[*a*]pyrene, PAHs from wood, coal, petroleum, other sources, and any source, in each job held. Odds ratios (ORs) for prostate cancer risk associated with lifetime PAHs exposure, adjusted for age, ancestry, education, lifestyle and occupational factors, and 95% confidence intervals (CI), were estimated using unconditional logistic regression.

**Results:**

After restriction to probable and definite exposures, and application of a 5-year lag, no clear association emerged for any of the PAHs, although small excesses in risk were apparent with 5-year increments in exposure to PAHs from wood (OR = 1.06, 95%CI 0.95 to 1.18). While analyses by cancer aggressiveness suggested no major differences, some elevated risk of high-grade cancer was observed for exposure to PAHs from wood (OR = 1.37, 95%CI 0.65 to 2.89), frequently occurring among firefighters.

**Conclusion:**

Findings provide weak support for an association between occupational exposure to PAHs from wood and prostate cancer risk.

**Supplementary Information:**

The online version contains supplementary material available at 10.1186/s12940-021-00751-w.

## Introduction

In the next two decades, around 11 million news cases of prostate cancer will be diagnosed worldwide [1]. The rising prostate cancer burden prompts for the identification of modifiable risk factors and primary preventive interventions [2]. Some thirty percent of prostate cancers are aggressive, but health issues associated with diagnosis and treatment of the non aggressive form of the disease remain of important significance.

The descriptive epidemiology of cancer of the prostate, a hormone-dependant organ [3], strongly suggests that non-genetic influences are at play [4–6]; genetic factors would only explain about one third of familial risk. An increasing attention is paid to the role of environmental exposures, some of which showing deleterious toxicological effects such as hormone-disruption or genotoxicity [7, 8]. Polycyclic aromatic hydrocarbons (PAHs) are a family of over 100 chemicals, generally found in complex mixtures occurring naturally in the environment or resulting from the incomplete combustion or pyrolysis of various organic materials [9]. In the general population, exposure to PAHs mostly occurs through tobacco smoking and from the cooking of food at high temperatures. The workplace remains a major source of PAHs exposure and several industries entailing high exposure to mixtures of PAHs are known or suspected carcinogens to humans [10].

PAHs’ ability to form DNA adducts through their ultimate metabolites interactions with DNA [11] or their antiestrogenic properties [12] may be involved in the initiation or promotion of prostate cancer. Benzo[*a*]pyrene is the sole PAH having been classified to date as carcinogenic to humans by the International Agency for Research on Cancer [10]. Only a handful of studies have examined the link between occupational exposure to PAHs and prostate cancer risk, with inconclusive findings. A cohort study including 1,386 incident cases investigating this association reported null findings with regards to overall exposure to PAHs [13]. Three small case–control studies have classified PAHs exposure according to their material sources [14–16]. One reported no association with ever exposure to PAHs [15], while positive associations with lower levels of exposure to benzo[*a*]pyrene [16], PAHs from coal [14, 16] and PAHs from other sources [16] emerged in the two others. No previous study has considered prostate cancer aggressiveness or detection issues that may obscure associations with exposure.

We previously reported that some industries (i.e., forestry and logging) and occupations (i.e., firefighters, gasoline station attendants) were at higher risk of prostate cancer [17]. By pointing to a potential deleterious role of occupational exposure to PAHs, these findings motivated the current investigation. We present here the largest, and one of the rare epidemiologic studies to date, evaluating the role of occupational exposure to PAHs overall, and according to various sources, in prostate cancer risk.

## Methods

### Study design

The present work emanates from the Prostate Cancer & Environment Study (PROtEuS), a large population-based case–control study conducted in Montreal, Canada in 2005–2012, and specifically designed to evaluate the role of occupational exposures. Details have been reported elsewhere [18, 19]. In order to comply with institutional regulations and insure comprehensive population coverage at recruitment, the study base was restricted to men who referred or would be expected to refer to a French hospital for a prostate cancer diagnosis. All subjects were residents of the Greater Montreal area. Incident prostate cancer cases, aged ≤ 75 years, histologically-confirmed, were identified through Montreal hospitals serving the French-speaking population, thereby covering 80% of all prostate cancer diagnoses in the area during the study period. Concurrently, controls were randomly selected from the continually-updated electoral list of French-speaking men, and frequency-matched to cases on age (± 5 years). Overall, 79% of cases (*n* = 1,937) and 56% of eligible controls (*n* = 1,994) agreed to participate in the study. Refusals (86%) were the main reason for non-participation. Ethics committees of all participating institutions approved the study protocol and all subjects provided written informed consent.

### Data collection

In-person interviews, conducted mostly at the home of participants, collected information on sociodemographic and lifestyle characteristics, and a complete occupational history. A few interviews (3% for cases, 4% for controls) were conducted with proxy respondents, mainly the spouses.

A detailed description was elicited for each job held for at least 2 years, including tasks and use of products, equipment, and protective measures. In addition, specialized questionnaires (*n* = 32), probing for specific task details, were administered for complex occupations lasting at least 5 years. Occupations and industries were coded according to Canadian classifications [20, 21].

Gleason scores, reflecting tumor grades, were extracted from pathology reports. These have been shown to be a good proxy for disease progression [22] and were used to define cancer severity. Tumor grades were recorded from diagnostic biopsies (main analyses) as well as from prostatectomy, when available.

#### Exposure assessment of PAHs

For each job, exposure to 313 agents, including PAHs, was assigned. PAH assessments included benzo[*a*]pyrene, as well as other PAHs grouped according to their sources, i.e., wood, petroleum, coal, or other sources (such as resulting from the pyrolysis products of plastic, paint, rubber, food or other organic compounds). Exposure to PAHs from any source was derived by aggregating the information from individual sources.

The hybrid expert-based assessment approach, described previously [23], was used to assign exposures based on the 16,065 detailed job descriptions. By providing clearer guidelines for coding, the hybrid expert-based approach was recently developed to improve on efficiency, comprehensiveness and transparency of assignments, and greater coherence between experts [23]. Basically, it makes use of job-exposure profiles summarizing expert evaluations from previous studies, complemented with comments on coding specific exposure circumstances, which together with the subjects’ job descriptions, guide experts in their assignments.

For each exposure, experts provided three semi-quantitative indices of exposure: the degree of confidence that the exposure actually occurred in the job (possible, probable, definite), the frequency of exposure (< 5%, 5%-30%, > 30%-90%, > 90% of usual workweek) and the relative intensity of exposure (low, medium, high) with non-exposure representing levels encountered in the general environment. Two hygienists, blinded to the case–control status of participants, carried out independent assessments; conflicting codings were resolved by consensus.

Exposure to benzo[*a*]pyrene was automatically assigned when other specific exposures (assigned by experts) occurred, conditional on their concentration, reliability, and frequency of exposure within the job, according to an algorithm devised by the team. The main chemical generators of benzo[*a*]pyrene were asphalt, coal and wood combustion products, coal and wood soot, tar and pitch, creosote, diesel and leaded engine emissions, ink formulation oils, mineral-based oil and greases, rubber pyrolysis products, and textile oils.

### Statistical analysis

For each PAH exposure, frequency intervals were recoded according to their median values. The three intensity levels were transformed into values of 1, 5 and 25, respectively, to better reflect the quantitative levels underlying the hygienists’ assignments [24].

Several exposure metrics were used, built from probable or definite exposures, and applying a 5-year lag: (i) ever exposure; (ii) duration of exposure, summing all exposed job periods over the course of the career; and (iii) cumulative exposure (CE) calculated as follows:

$$\text{CE}=\sum_{i=1}^k\left(\text{I}_i\times \text{F}_i\times \text{D}_i\right)$$

where *i* represented the *i*^th^ job held, *k* was the total number of jobs held, I_*i*_ was the intensity of exposure in the job *i*, F_*i*_ the frequency of exposure in the job *i* and D_*i*_ the duration of the job *i* in years.

Odds ratios (ORs) and their corresponding 95% confidence intervals (CI) were calculated using unconditional logistic regression, modelling the association between exposure to each PAH and prostate cancer risk. Polytomous regression models were applied to assess risk according to cancer aggressiveness. Low-grade tumors (non-aggressive) were those with Gleason scores ≤ 6 or 7 [with 3 as primary and 4 as secondary pattern] while the high-grade ones (aggressive) were those with scores ≥ 8 or 7 [with 4 as primary and 3 as secondary pattern] [25]. Heterogeneity in risk between the two grades was investigated with the Wald test.

In all analyses, the reference category included men who had never been occupationally exposed to any of the PAHs under investigation, those whose assignments to PAHs across all jobs had only been judged as possible, and those who had been exposed to PAHs only within the 5 years preceding the diagnosis/interview.

The cumulative duration of exposure was expressed in terms of a 5-year range increase, after confirming linearity of the logit. We also categorized CE according to the 50^th^ and 75^th^ percentiles of the controls’ distributions as follows: ‘low exposure’ [< 50^th^ (excluding 0)], ‘medium exposure’ [50^th^ to 75^th^] and ‘high exposure’ for percentiles beyond the 75^th^. The medians of the CE categories were modelled as continuous variables allowing for the evaluation of dose–response relationships.

Sensitivity analyses were conducted to evaluate the robustness of our results: (1) restricting to controls who had been screened for prostate cancer in the two years preceding the interview, thereby reducing the likelihood of latent, undiagnosed cases, (2) applying a lag of 10 years; and (3) using other weights for the intensity of exposure (1,3,9 and 1,10,100 instead of 1,5,25).

We also explored the timing of occupational exposure to PAHs over the course of the participants’ careers—that is, whether the last job entailing exposure to PAHs had been held within the 10 years prior to the index date or further in the past.

All analyses were adjusted for factors that we identified as potential confounders with a causal directed acyclic graph: age at diagnosis/interview (continuous), ancestry (Sub-Saharan, Asian, French, Other European, Greater Middle Eastern, Latino, other), educational level (primary school or less, high school, college, university, other), alcohol drinking (drink-years), cigarette smoking (ever/never and cigarette-years), body mass index (in kg/m^2^: < 18.50, 18.50–24.99, 25.00–29.99, ≥ 30.00), ever consumption of fried or grilled food, cumulative occupational exposure to benzene (non-exposed referents and tertiles of exposure), ever farming and workplace environmental tobacco smoke (non-exposed referents and tertiles of exposure).

The SAS software (version 9.4; SAS Institute, Cary, North Carolina) was used.

## Results

In total, 1,924 prostate cancer cases and 1,989 controls contributed to the analyses. Most subjects were of French descent. Cases were more likely to have had primary school as their highest level of education. Seventy-six percent of controls had been screened for prostate cancer within 2 years of interview (Table 1).

**Table 1:** Selected characteristics of cases and controls, PROtEuS, Montreal, Canada, 2005–2012

	Prostate cancer cases	Controls
	*n* = 1,924	*n* = 1,989
	n (%)	n (%)
Sociodemographic characteristics		
*Age* (years)		
Mean ± sd	63.6 ± 6.8	64.8 ± 6.9
*Ancestry*		
Sub-Saharan	129 (6.7)	89 (4.5)
Asian	24 (1.3)	72 (3.5)
French	1439 (75.0)	1243 (62.5)
Other European	245 (12.5)	438 (22.0)
Greater Middle Eastern	45 (2.3)	99 (5.0)
Latino	29 (1.5)	31 (1.6)
Other	1 (0.1)	3(0.2)
Missing	12 (0.6)	14 (0.7)
*Educational level*		
Primary school of less	448 (23.3)	427 (21.4)
High school	571 (29.6)	576 (29.0)
College	312 (16.2)	374 (18.8)
University	588 (30.6)	610 (30.7)
Other	5(0.3)	2(0.1)
*Number of years since last prostate cancer screening (PSA and/or DRE)*		
≤ 2.00 years	1904 (99.0)	1509 (75.9)
2.01 – 5.00 years	1 (0.1)	152 (7.5)
> 5.00 years	0 (0.0)	81 (4.1)
Never screened	3 (0.2)	191 (9.6)
Do not know if ever screened	2 (0.1)	29 (1.5)
Had screening but do not know when	14 (0.6)	27 (1.4)

*Abbreviations*: *PSA* Prostate specific antigen, *DRE* Digital rectal examination

Additional file 1  lists some of the main occupations in which PAHs were assigned, according to exposure dimensions.

PAH-exposed occupations included firefighters, boilermakers, platers and structural-metal workers, timber cutting and related occupations, and printing press occupations. There was often concomitant exposure to benzo[*a*]pyrene and other PAHs. Exposure to PAHs from wood mostly occurred at high intensity in firefighting occupations. Occupations in labouring and other elemental work (not elsewhere classified (n.e.c)), and timber cutting and related occupations were more occasionally exposed and at low intensity of exposure. Firefighting occupations nearly always entailed occupational exposure to PAHs, including benzo[*a*]pyrene, PAHs from wood, petroleum and other sources, but had no exposure to PAHs from coal.

A larger range of occupations were exposed to PAHs from petroleum, including all aircraft mechanics and repairmen, and almost all industrial, farm and construction machinery mechanics and repairmen. Knitting occupations and printing press occupations were the most highly exposed occupations to PAHs from this source.

Exposure to PAHs from other sources also frequently occurred among motor-vehicle mechanics and repairmen, and sometimes among foremen of mechanics and repairmen (n.e.c) and boilermakers, platers and structural metal-workers.

Exposures to PAHs were weakly to moderately correlated with one another (see Additional file 2). The strongest correlations were found between PAHs from other sources and PAHs from petroleum (*r* = 0.33) and between PAHs from other sources and PAHs from wood (*r* = 0.27).

Benzo[*a*]pyrene exposure occurred in most of jobs exposed to PAHs from wood (88% of 143 exposed jobs) and to PAHs from coal (87% of 172 exposed jobs). Contrastingly, jobs exposed to PAHs from other sources and to PAHs from petroleum less often entailed benzo[*a*]pyrene exposure (33% of 384 exposed jobs and 23% of 2,399 exposed jobs, respectively).

Associations between occupational exposure to the different PAHs and risk of overall prostate cancer (notwithstanding tumor aggressiveness) are presented in Table 2. No clear association emerged for any of the PAHs and for any of the exposure metrics (ever, duration or cumulative exposure). However, a weak increase in risk was found in association with PAHs from wood for ever exposure (OR = 1.24, 95%CI 0.79 to 1.95) and duration (OR = 1.06, 95%CI 0.96 to 1.18, per 5-year increment), and in the highest category of cumulative exposure (OR = 1.54, 95%CI 0.60 to 3.92), albeit without a dose–response pattern (p for trend = 0.32).

**Table 2:** Association Between Occupational Exposure to PAHs and Prostate Cancer Risk, PROtEuS, 2005–2012

Exposure		Prostate cancer		
	n Controls	n Cases	OR^**a**^	95%CI
*PAHs from any source*				
Never exposed	1053	1009	1.00	Ref
Ever exposed	573	560	1.00	(0.85, 1.18)
Duration (per 5-year increment)	573	560	1.00	(0.97, 1.03)
Cumulative exposure				
0.01—300.00	303	293	0.99	(0.81, 1.20)
300.01—1049.66	125	130	1.03	(0.78, 1.37)
> 1049.66	143	137	1.01	(0.76, 1.33)
p for trend			0.93	
*Benzo[a]pyrene*				
Ever exposed	188	182	1.00	(0.78, 1.26)
Duration (per 5-year increment)	188	182	0.97	(0.92, 1.03)
Cumulative exposure				
0.01—226.95	94	103	1.12	(0.81, 1.55)
226.96—588.00	42	42	1.03	(0.64, 1.65)
> 588.00	52	37	0.73	(0.45, 1.20)
p for trend			0.25	
*PAHs from petroleum*				
Ever exposed	516	514	1.01	(0.85, 1.19)
Duration (per 5-year increment)	516	514	1.01	(0.97, 1.04)
Cumulative exposure				
0.01—240.50	266	278	1.06	(0.86, 1.30)
240.51—901.00	120	115	0.89	(0.67, 1.20)
> 901.00	130	121	1.02	(0.76, 1.37)
p for trend			0.93	
*PAHs from wood*				
Ever exposed	42	50	1.24	(0.79, 1.95)
Duration (per 5-year increment)	42	50	1.06	(0.95, 1.18)
Cumulative exposure				
0.01—450.00	21	24	1.13	(0.60, 2.16)
450.01—1732.50	10	8	0.94	(0.33, 2.66)
> 1732.50	11	18	1.54	(0.60, 3.92)
p for trend			0.32	
*PAHs from coal*				
Ever exposed	57	57	0.97	(0.66, 1.44)
Duration (per 5-year increment)	57	57	0.95	(0.87, 1.04)
Cumulative exposure				
0.01—142.50	30	36	1.15	(0.68, 1.95)
142.51—546.25	12	14	1.18	(0.52, 2.70)
> 546.25	15	7	0.46	(0.18, 1.17)
p for trend			0.12	
*PAHs from other sources*				
Ever exposed	88	91	0.95	(0.68, 1.31)
Duration (per 5-year increment)	88	91	1.01	(0.94, 1.08)
Cumulative exposure				
0.01—200.00	44	41	0.89	(0.56, 1.41)
200.01—725.00	21	19	0.73	(0.37, 1.42)
> 725.00	23	31	1.19	(0.62, 2.30)
p for trend			0.59	

*Abbreviations*: *OR* Odds ratio, *PAHs* Polycyclic aromatic hydrocarbons, *CI* Confidence interval

^a^OR adjusted for age, ancestry, education, alcohol drinking, cigarette smoking, body mass index, consumption of fried or grilled food, occupational exposure to benzene, farming, and workplace environmental tobacco smoke

Firefighters were the occupational group the most often exposed to PAHs from wood. The OR for prostate cancer among the 40 firefighters exposed to PAHs from this source was 1.27 (95%CI 0.61 to 2.67).

Table 3 shows associations between occupational exposure to PAHs and prostate cancer risk, by tumor aggressiveness. Risk estimates were generally close to unity for all of the PAHs studied. For aggressive prostate cancers, ORs of 1.28, 1.57 and 1.73 were found for the middle category of cumulative exposure to PAHs from any source, benzo[*a*]pyrene and PAHs from wood, respectively, but there was no exposure–response pattern. Some weak elevation in risk of high-grade cancers (OR = 1.37, 95%CI 0.65 to 2.89) was apparent for ever exposure to PAHs from wood, based on 13 exposed cases. The ORs per 5-year increment of duration of exposure to PAHs from wood were 1.07 (95%CI 0.96 to 1.20) and 1.01 (95%CI 0.85 to 1.21), for low- and high-grade tumors, respectively. For low-grade cancers, an OR of 1.74 (95%CI 0.67 to 4.56) emerged for the highest cumulative exposure category, without apparent gradient in risk (p for trend= 0.19).

**Table 3:** Association Between Occupational Exposure to PAHs and Prostate Cancer Risk, by Tumor Aggressiveness, PROtEuS, 2005–2012

Exposure		Low-grade prostate cancer *n* = 1,488			High-grade prostate cancer *n* = 436		
	n Co	n Ca	OR^**a**^	95%CI	n Ca	OR^**a**^	95%CI
*PAHs from any source*							
Never exposed to any source	1053	803	1.00	Ref	211	1.00	Ref
Ever exposed	573	420	0.97	(0.81, 1.17)	142	1.08	(0.83, 1.42)
Duration (per 5-year increment)	573	420	0.99	(0.96, 1.03)	142	1.01	(0.97, 1.07)
Cumulative exposure							
0.01—300.00	303	221	0.96	(0.78, 1.19)	73	1.06	(0.78, 1.46)
300.01—1049.66	125	91	0.95	(0.70, 1.30)	39	1.28	(0.84, 1.95)
> 1049.66	143	108	1.02	(0.75, 1.39)	30	0.93	(0.58, 1.47)
p for trend			0.86			0.79	
*Benzo[a]pyrene*							
Ever exposed	188	136	0.98	(0.74, 1.30)	46	1.08	(0.71, 1.64)
Duration (per 5-year increment)	188	136	0.96	(0.91, 1.02)	46	1.00	(0.93, 1.09)
Cumulative exposure							
0.01—226.95	94	82	1.17	(0.83, 1.66)	22	0.97	(0.57, 1.67)
226.96—588.00	42	27	0.86	(0.51, 1.46)	15	1.57	(0.81, 3.04)
> 588.00	52	28	0.68	(0.40, 1.17)	9	0.85	(0.38, 1.90)
p for trend			0.16			0.85	
*PAHs from petroleum*							
Ever exposed	518	390	1.01	(0.84, 1.22)	126	1.03	(0.78, 1.36)
Duration (per 5-year increment)	518	390	1.00	(0.97, 1.04)	126	1.02	(0.97, 1.07)
Cumulative exposure							
0.01—240.50	266	209	1.04	(0.83, 1.30)	69	1.12	(0.81, 1.55)
240.51—901.00	120	87	0.89	(0.65, 1.22)	28	0.90	(0.56, 1.44)
> 901.00	130	93	1.05	(0.77, 1.45)	28	0.93	(0.58, 1.49)
p for trend			0.92			0.63	
*PAHs from wood*							
Ever exposed	42	37	1.11	(0.65, 1.89)	13	1.37	(0.65, 2.89)
Duration (per 5-year increment)	42	37	1.07	(0.96, 1.20)	13	1.01	(0.85, 1.21)
Cumulative exposure							
0.01—450.00	21	16	1.02	(0.50, 1.08)	8	1.45	(0.59, 3.58)
450.01—1732.50	10	4	0.64	(0.19, 2.25)	4	1.73	(0.43, 6.95)
< 1732.50	11	17	1.74	(0.67, 4.56)	1	0.50	(0.05, 4.72)
p for trend			0.19			0.52	
*PAHs from coal*							
Ever exposed	57	40	0.92	(0.59, 1.44)	17	1.09	(0.58, 2.03)
Duration (per 5-year increment)	57	40	0.93	(0.84, 1.03)	17	0.99	(0.87, 1.12)
Cumulative exposure							
0.01—142.50	30	26	1.11	(0.63, 1.96)	10	1.27	(0.58, 2.81)
142.51—546.25	12	10	1.16	(0.47, 2.83)	4	1.13	(0.33, 3.85)
> 546.25	15	4	0.36	(0.11, 1.12)	3	0.71	(0.19, 2.61)
p for trend			0.09			0.62	
*PAHs from other sources*							
Ever exposed	88	69	0.88	(0.60, 1.29)	22	0.99	(0.57, 1.75)
Duration (per 5-year increment)	88	69	1.02	(0.94, 1.09)	22	0.97	(0.87, 1.09)
Cumulative exposure							
0.01—200.00	44	29	0.83	(0.50, 1.37)	12	1.09	(0.54, 2.19)
200.01—725.00	21	15	0.75	(0.37, 1.53)	4	0.66	(0.21, 2.07)
> 725.00	23	25	1.18	(0.59, 2.38)	6	1.14	(0.39, 3.34)
p for trend			0.55			0.97	

*Abbreviations*: *PAHs* Polycyclic aromatic hydrocarbons, *CI* Confidence interval, *OR *Odds ratio, *Ca* Cases, *Co* Controls

^a^OR adjusted for age, ancestry, education, alcohol drinking, cigarette smoking, body mass index, consumption of fried or grilled food, occupational exposure to benzene, farming, and workplace environmental tobacco smoke

We investigated whether risks varied according to the timing of exposure to the various PAHs based on the last exposed job. There were marginal differences in risk estimates between recent (≤ 10 years before index date) and remote (˃ 10 years) exposure to PAHs from any source, from petroleum and from other sources (data not shown). There were, however, suggestions of excess risks for remote exposure to benzo[*a*]pyrene (OR = 1.22, 95%CI 0.90 to 1.64), PAHs from wood (OR = 1.27, 95%CI 0.75 to 2.16) and PAHs from coal (OR = 1.28, 95%CI 0.78 to 2.09). Results were similar across aggressiveness categories, other than for PAHs from wood, where remote exposure appeared to be associated with some greater risk of high-grade cancers (OR = 1.54, 95%CI 0.70 to 3.39, based on 12 exposed cases), as compared to low-grade ones (OR = 1.18, 95%CI 0.66 to 2.08, based on 31 exposed cases).

In analyses focusing on firefighters exposed to PAHs from wood, the odds of low-grade cancer was 1.47 (95%CI 0.69 to 3.16) while that of high-grade cancer was 0.54 (95%CI 0.11 to 2.59).

It has been suggested that environment-induced prostate cancers could occur more often among men aged over 55 years at diagnosis, as younger cases would be more likely to result from genetic causes [26]. There was no interaction between age and PAHs exposures in the present study, but the number of subjects diagnosed at a younger age was low, which may have hampered our ability to detect an effect modification by age.

Tumor aggressiveness was ascertained from Gleason scores at diagnostic biopsy (all cases, main analysis). For 903 cases (47%) we were able to follow-up, we also had information on the Gleason score derived from prostatectomy. Prioritizing prostatectomy scores over those from biopsies led to a confirmation of 94% and 85% of non-aggressive and aggressive cancers, respectively, according to our pre-established cut-points. Based on this, no major changes in risk estimates for associations with PAHs were observed compared with those based on biopsy results only. A slight excess in risk could still be observed in association with cumulative exposure to PAHs from wood (data not shown).

Sensitivity analyses excluding controls not recently screened for prostate cancer (see Additional files 3  and 4) or applying alternative weights to intensity categories (data not shown) generated results consistent with our main findings.

## Discussion

Our results provide weak support to the hypothesis that occupational exposure to PAHs is associated with prostate cancer risk. Small excesses in risk were apparent in relation with duration and the highest levels of exposure to PAHs from wood, while remote exposures appeared to be especially associated with high-grade cancers.

Benzo[*a*]pyrene and several industries entailing high exposure to mixtures of PAHs containing benzo[*a*]pyrene have been classified as carcinogenic to humans for sites other than the prostate [10]. Very few studies to date have examined the role of specific occupational exposures in prostate cancer risk, including PAHs, precluding drawing definitive conclusions. Nevertheless, the sole cohort study and three case–control studies investigating this association are informative as exposure to PAHs was based on strong exposure assessment protocols and potential confounding was accounted for, although most of these relied on small samples [13–16].

As in our study, all of the case–control studies of prostate cancer conducted to date have examined the role of PAHs from any source, as well as according to their source material [14–16]. While there is, to our knowledge, no evidence that the carcinogenic potential of PAHs would differ according to their source, the latter can reflect, as is the case here for PAHs from wood, exposure circumstances within specific occupations (e.g. firefighters). By contrast, the one cohort study of prostate cancer assessing exposure to PAHs did not distinguish their sources [13].

In our study, other than for the weak signal for an association with PAHs from wood, there was generally no association between exposure to benzo[*a*]pyrene and other PAHs, and overall prostate cancer risk. An American case–control study observed no association with ever exposure to the same PAHs grouping than us, although benzo[*a*]pyrene was not examined [15]. While some increases in risk of overall prostate cancer have been linked to low exposure levels to benzo[*a*]pyrene [14, 16], PAHs from coal [14, 16] and PAHs from other sources [16], results for high exposure levels were consistent with no association with these PAHs [14, 16], but also those from petroleum, wood [16] and more globally to PAHs from any source [13, 14, 16].

Our study is the first to investigate associations between PAHs and prostate cancer aggressiveness, defined by tumor histological grade. Previous work suggests that aggressive cancers may have a different set of risk factors, including occupational [19], than their non-aggressive counterpart [27], and that they may have distinct aetiologies [28]. No formal statistical heterogeneity in risk emerged between low- and high-grade cancers, possibly reflecting a lack of statistical power to detect differences (there were fewer high-grade tumors and CIs were wider). In one previous study assessing occupational exposure to PAHs and the degree of spreading of the tumor at diagnosis, no association emerged [13].

Exposure to PAHs from wood mostly occurred in firefighting occupations. A higher prostate cancer screening rate in firefighters, as compared to other workers, could have resulted in a positive association with exposure in this group of workers. There is indeed evidence that screening patterns differ according to occupation [29]. However, there was no evidence in our study that firefighters were screened more frequently than other workers (data not shown). Firefighting occupations have been reported at elevated risk of prostate cancer in several studies [30] including in the present study population [17]. Exposure to PAHs, which was not measured in these studies, might explain the excesses in risk, and/or possibly, nightshift work. However, we recently documented the absence of an association between nightshift work and prostate cancer in the PROtEuS study [18], and consequently have not adjusted for this in our models.

Incomplete wood combustion is an important source of benzo[*a*]pyrene but also of other PAHs such as dibenz[a,h]anthracene, a probable human carcinogen [10]. We found no association with benzo[*a*]pyrene or with other groups of PAHs that likely contain this chemical.

Cigarette smoking was weakly correlated with occupational exposure to PAHs (data not shown). The strongest correlation was between cigarette smoking and PAHs from petroleum (*r* = 0.10). All correlation coefficients between fried or grilled food consumption and PAHs were close to the null value. These observations argue against a potential over-adjustment for these factors.

There is evidence that PAHs could be genotoxic to the prostate gland through the local formation of PAH-DNA adducts [31]. An interaction between high occupational exposure to PAHs and the glutathione S-transferase polymorphism (GSTP1), a detoxifying gene, has been shown to be associated with an increased prostate cancer risk [15]. Variants of this gene are known to be involved in the early process of carcinogenesis in prostate gland [32]. Others also reported that the combination of non-functionnal GSTP polymorphisms [33] or the presence of GSTP1 methylation in prostate tissue [34] were predisposing factors for prostate cancer.

This study has some limitations. There inevitably was exposure misclassification, probably attenuating risk estimates. While this is the largest study to date, based on the number of exposed cases, conducted on this subject, it may have been underpowered to detect weaker associations, especially in sub-group analyses. Exposure assessment was not conducted for jobs lasting less than 2 years, although this resulted in less than 4% of work-years, on average, not being covered [35]. Finally, response rates, albeit typical to those encountered in similar community-based studies [36], were good for cases but moderate for controls. Comparisons of area-based indicators (unemployment, education, % of recent immigrants, income) between participants and non-participants showed little differences, reassuring against strong selection bias.

The study also builds on several strengths. The statistical power calculated when designing PROtEuS was quite good, allowing for the detection of moderate associations. Exposure assessment was conducted by expert review, which enables consideration of job-specific details and idiosyncrasies. This has long been considered to be the reference approach for community-based retrospective studies [37, 38]. We further improved on the transparency of the coding by applying a hybrid approach to guide the experts [23]. PAHs exposures were studied together as well as according to their material source, thereby capturing more specific exposure circumstances. Though residual confounding remains possible, we controlled for factors and occupational agents suspected to be associated with prostate cancer. Other factors included in the models are those that might also include PAHs exposure, such as cigarette smoking and consumption of fried or grilled food. We investigated their correlation with PAHs exposures using Spearman correlation and Kendall's tau-b correlation (data not shown). Cigarette smoking was weakly correlated with occupational exposure to PAHs. The strongest correlation was between cigarette smoking and PAHs from petroleum (*r* = 0.10). All correlation coefficients between fried or grilled food consumption and PAHs were close to the null value. These observations argue against a potential over-adjustment by these factors.

Sensitivity analyses showed consistent results with those from the main analyses. Unlike other previous investigations, we had information on prostate cancer grade, enabling to evaluate associations by cancer aggressiveness. Histology from biopsy categorised into Gleason score is considered to be the ‘gold standard’ for the diagnosis of prostate cancer [39], and is commonly used to stratify the risk of men and to choose their consecutive treatment. Radical prostatectomy, when indicated, is often deferred for several weeks after biopsy [40]. In some cases, there is a discrepancy between biopsy and prostatectomy Gleason scores, characterized by the upgrading or under-grading of prostate cancer on biopsies. Here, prioritizing findings from prostatectomies resulted in classification changes for 6% of the initial non-aggressive cancer cases and 15% of the aggressive ones, but results remained similar to the main findings. And finally, we had information on prostate cancer screening practices and accounted for these in our analyses. Our study population is quite unique in that respect. While there was no prostate cancer screening program in place at the time of study, screening tests were often part of annual medical routine exams in this population, which has universal and free access to healthcare. The lack of considering detection issues in epidemiological studies of prostate cancer has been recognized as a salient problem that may have obscured associations and hampered progress in identifying risk factors for this cancer [41, 42].

## Conclusion

Overall, our results provide weak support to the hypothesis that occupational exposure to PAHs is associated with prostate cancer risk.

## Supplementary Information

**Additional file 1: Table S1.** Most Frequent* Occupations Classified as Having Probable or Definite Occupational Exposure to Polycyclic Aromatic Hydrocarbons (PAHs), PROtEuS, Montreal, 2005-2012.

**Additional file 2: Figure. **Spearman Correlation’s Coefficients Between Cumulative Exposure to Polycyclic Aromatic Hydrocarbons From Various Sources Among Controls, PROtEuS, Montreal, Canada, 2005-2012.

**Additional file 3: Table S2.** Association Between Occupational Exposure to PAHs and Prostate Cancer Risk, Restricting Controls to Men Screened for Prostate Cancer in the Two Years Prior to Interview, PROtEuS, Montreal, Canada, 2005-2012.

**Additional file 4: Table S3.** Association Between Occupational Exposure to PAHs and Prostate Cancer Risk, by Tumor Aggressiveness, Restricting Controls to Men Screened for Prostate Cancer in the Two Years Prior to Interview, PROtEuS, Montreal, Canada, 2005-2012.

## Data Availability

The data that support the findings of this study are available from the corresponding author upon request that meet confidentiality agreement with study subjects.
