# Supplementary material for: Occupational exposure to polycyclic aromatic hydrocarbons and risk of prostate cancer
Source: Environ Health. 2021 Jun 21;20:71. doi: 10.1186/s12940-021-00751-w (PMC8218525; doi:10.1186/s12940-021-00751-w)
Supplement: Supplementary file 1 — Additional file 1: Table S1. Most Frequent* Occupations Classified as Having Probable or Definite Occupational Exposure to Polycyclic Aromatic Hydrocarbons (PAHs), PROtEuS, Montreal, 2005-2012. [file 12940_2021_751_MOESM1_ESM.docx]

**Additional file 1: Table S1.** Most Frequent* Occupations Classified as Having Probable or Definite Occupational Exposure to Polycyclic Aromatic Hydrocarbons (PAHs), PROtEuS, Montreal, 2005-2012

| **PAHs from any source** |  | | **Most frequent exposure coding** | | |
| --- | --- | --- | --- | --- | --- |
| **CCDO.Occupations** | **n jobs** | **%** | **Reliability^a^** | **Frequency^b^** | **Intensity^c^** |
| 8582.Aircraft mechanics and repairmen | 29 | 100 | 3 | 3 | 1 |
| 8584.Industrial, farm and construction machinery mechanics and repairmen | 176 | 99 | 3 | 3 | 1 |
| 8711.Excavating, grading and related occupations | 39 | 97 | 3 | 3 | 1 |
| 6111.Fire-fighting occupations | 65 | 95 | 3 | 2 | 3 |
| 8337.Boilermakers, platers and structural-metal workers | 24 | 92 | 2 | 3 | 2 |
| 8526.Inspecting and testing occupations, equipment repair, n.e.c | 21 | 90 | 3 | 3 | 1 |
| 8271.Knitting occupations | 30 | 97 | 3 | 3 | 3 |
| 8315.Machine-tool operating occupations | 45 | 96 | 3 | 3 | 1 |
| 8313.Machinist and machine-tool setting-up occupations | 141 | 95 | 3 | 3 | 1 |
| 8581.Motor-vehicle mechanics and repairmen | 215 | 96 | 3 | 3 | 1 |
| 9533.Stationary engine and auxiliary equipment operating and maintaining occupations | 28 | 96 | 3 | 2 | 1 |
| 5145.Service station attendants | 27 | 93 | 3 | 2 | 1 |
| 8337.Boilermakers, platers and structural-metal workers | 24 | 92 | 2 | 3 | 2 |
| 8526.Inspecting and testing occupations, equipment repair, n.e.c | 21 | 90 | 3 | 3 | 1 |
| 8589.Other mechanics and repairmen, n.e.c | 27 | 89 | 3 | 3 | 1 |
| 8334.Metalworking-machine operators, n.e.c | 38 | 89 | 3 | 3 | 1 |
| 8580.Foremen: mechanics and repairmen, n.e.c | 76 | 86 | 3 | 3 | 1 |
| 7513.Timber cuttings and related occupations | 58 | 81 | 3 | 3 | 2 |
| 8533.Electrical and related equipment installing and repairing occupations, n.e.c | 84 | 73 | 3 | 3 | 1 |
| 9512.Printing press occupations | 63 | 71 | 3 | 3 | 3 |
| 9918.Occupations in labouring and other elemental work, N.E.C | 25 | 60 | 3 | 2 | 1 |
| 8335.Welding and flame cutting occupations | 161 | 59 | 2 | 3 | 2 |
| 9311.Hoisting occupations, n.e.c | 37 | 57 | 3 | 2 | 1 |
| 9113.Air transport operating support occupations | 21 | 57 | 3 | 3 | 1 |
| **Benzo[*a*]pyrene** |  | | | | |
| **CCDO.Occupations** | **n jobs** | **%** | **Reliability** | **Frequency** | **Intensity** |
| 6111.Fire-fighting occupations | 65 | 95 | 3 | 2 | 2 |
| 8337.Boilermakers, platers and structural-metal workers | 24 | 88 | 2 | 3 | 1 |
| 7513.Timber cutting and related occupations | 58 | 59 | 2 | 3 | 1 |
| 9512.Printing press occupations | 63 | 54 | 3 | 3 | 1 |
| 8335.Welding and flame cutting occupations | 161 | 49 | 2 | 3 | 1 |
| 8271.Knitting occupations | 30 | 43 | 2 | 3 | 1 |
| 9918.Occupations in labouring and other elemental work, n.e.c | 25 | 64 | 2 | 2 | 2 |
| 8584.Industrial, farm and construction machinery mechanics and repairmen | 176 | 32 | 3 | 3 | 1 |
| 8315.Machine-tool operating occupations | 45 | 27 | 3 | 3 | 1 |
| 7111.General farmers | 37 | 22 | 2 | 2 | 1 |
| 8580.Foremen: mechanics and repairmen, n.e.c | 76 | 21 | 3 | 3 | 1 |
| **PAHs from petroleum** |  |  |  |  |  |
| **CCDO.Occupations** | **n jobs** | **%** | **Reliability** | **Frequency** | **Intensity** |
| 8582.Aircraft mechanics and repairmen | 29 | 100 | 3 | 3 | 1 |
| 8584.Industrial, farm and construction machinery mechanics and repairmen | 176 | 99 | 3 | 3 | 1 |
| 8711.Excavating, grading and related occupations | 39 | 97 | 3 | 3 | 1 |
| 8271.Knitting occupations | 30 | 97 | 3 | 3 | 3 |
| 5145.Service station attendants | 27 | 93 | 3 | 2 | 1 |
| 8526.Inspecting and testing occupations, equipment repair, n.e.c | 21 | 90 | 3 | 3 | 1 |
| 8581.Motor-vehicle mechanics and repairmen | 215 | 96 | 3 | 3 | 1 |
| 8315.Machine-tool operating occupations | 46 | 96 | 3 | 3 | 1 |
| 8337.Boilermakers, platers and structural-metal workers | 24 | 92 | 2 | 3 | 2 |
| 8313.Machinist and machine-tool setting-up occupations | 141 | 95 | 3 | 3 | 1 |
| 8334.Metalworking-machine operators, n.e.c | 38 | 89 | 3 | 3 | 1 |
| 9533.Stationary engine and auxiliary equipment operating and maintaining occupations | 28 | 89 | 3 | 2 | 1 |
| 6111.Fire-fighting occupations | 65 | 85 | 3 | 2 | 1 |
| 8580.Foremen: mechanics and repairmen, n.e.c | 76 | 83 | 3 | 3 | 1 |
| 8592.Marine craft fabricating, assembling and repairing occupations | 23 | 83 | 3 | 3 | 1 |
| 8589.Other mechanics and repairmen , n.e.c | 27 | 89 | 3 | 3 | 1 |
| 7513.Timber cuttings and related occupations | 58 | 76 | 3 | 3 | 2 |
| 9512.Printing press occupations | 63 | 71 | 3 | 3 | 3 |
| 8533.Electrical and related equipment installing and repairing occupations, n.e.c | 84 | 70 | 3 | 3 | 1 |
| 9113.Air transport operating support occupations | 21 | 57 | 3 | 3 | 1 |
| 9311.Hoisting occupations, n.e.c | 37 | 57 | 3 | 2 | 1 |
| 9113.Air transport operating support occupations | 21 | 57 | 3 | 3 | 1 |
| 9918.Occupations in labouring and other elemental work, n.e.c | 25 | 56 | 2 | 1 | 1 |
| 8335.Welding and flame cutting occupations | 161 | 52 | 2 | 3 | 2 |
| **PAHs from wood** |  |  |  |  |  |
| **CCDO.Occupations** | **n jobs** | **%** | **Reliability** | **Frequency** | **Intensity** |
| 6111.Fire-fighting occupations | 65 | 95 | 3 | 2 | 3 |
| 9918.Occupations in labouring and other elemental work, n.e.c | 25 | 20 | 2 | 1 | 1 |
| 7513.Timber cutting and related occupations | 58 | 10 | 2 | 3 | 1 |
| **PAHs from coal** |  |  |  |  |  |
| **CCDO.Occupations** | **n jobs** | **%** | **Reliability** | **Frequency** | **Intensity** |
| 9918.Occupations in labouring and other elemental work, n.e.c | 25 | 24 | 2 | 2 | 2 |
| 8781.Carpenters and related occupations | 152 | 14 | 3 | 1 | 1 |
| 8782.Brick and stone masons and tile setters | 45 | 13 | 3 | 1 | 1 |
| 8797.Occupation in labouring and other elemental work, other construction trades | 74 | 11 | 2 | 1 | 1 |
| **PAHs from other sources** |  |  |  |  |  |
| **CCDO.Occupations** | **n jobs** | **%** | **Reliability** | **Frequency** | **Intensity** |
| 6111.Fire-fighting occupations | 65 | 88 | 3 | 2 | 3 |
| 8581.Motor-vehicle mechanics and repairmen | 215 | 60 | 3 | 2 | 2 |
| 8580.Foremen: mechanics and repairmen, n.e.c | 76 | 28 | 2 | 2 | 1 |
| 8337.Boilermakers, platers and structural-metal workers | 24 | 25 | 3 | 1 | 3 |
| 8335.Welding and flame cutting occupations | 161 | 22 | 2 | 2 | 2 |
| 8584.Industrial, farm and construction machinery mechanics and repairmen | 176 | 20 | 3 | 3 | 1 |

*Number of jobs > 20 and percentage of jobs exposed to: PAHs from any sources ≥ 50%, benzo[*a*]pyrene ≥ 20%, PAHs from petroleum ≥ 50%, PAHs from wood ≥ 10%, PAHs from coal ≥ 10%, PAHs from other sources ≥ 20%.

^a^Reliability (1=possible, 2=probable, 3= definite), ^b^Frequency, (<5%, 5%-30%, >30%-90%, >90% of usual workweek), ^c^Intensity (1=low, 2=medium, 3=high, with low representing a level above the background environmental level).

N jobs: total number of coded jobs, %, percentage of jobs exposed; CCDO, Canadian Classification and Dictionary of Occupations, n.e.c, not elsewhere classified; PROtEuS, Prostate Cancer & Environment study.
