# Supplementary material for: Occupational exposure to polycyclic aromatic hydrocarbons and risk of prostate cancer
Source: Environ Health. 2021 Jun 21;20:71. doi: 10.1186/s12940-021-00751-w (PMC8218525; doi:10.1186/s12940-021-00751-w)
Supplement: Supplementary file 2 — Additional file 2: Figure. Spearman Correlation’s Coefficients Between Cumulative Exposure to Polycyclic Aromatic Hydrocarbons From Various Sources Among Controls, PROtEuS, Montreal, Canada, 2005-2012. [file 12940_2021_751_MOESM2_ESM.docx]

**Additional file 2**

| PAHs  from petroleum | PAHs  from petroleum |  |  |  |
| --- | --- | --- | --- | --- |
| PAHs  From wood | 0.09 | PAHs  from wood |  |  |
| PAHs  from coal | 0.11 | 0.12 | PAHs  from coal |  |
| PAHs  from other sources | 0.33 | 0.27 | 0.02 | PAHs from  other sources |

Abbreviations: PAHs: polycyclic aromatic hydrocarbons

**Figure:** Spearman Correlation’s Coefficients Between Cumulative Exposure to Polycyclic Aromatic Hydrocarbons From Various Sources Among Controls, PROtEuS, Montreal, Canada, 2005-2012
