# Supplementary material for: Occupational exposure to polycyclic aromatic hydrocarbons and risk of prostate cancer
Source: Environ Health. 2021 Jun 21;20:71. doi: 10.1186/s12940-021-00751-w (PMC8218525; doi:10.1186/s12940-021-00751-w)
Supplement: Supplementary file 3 — Additional file 3: Table S2. Association Between Occupational Exposure to PAHs and Prostate Cancer Risk, Restricting Controls to Men Screened for Prostate Cancer in the Two Years Prior to Interview, PROtEuS, Montreal, Canada, 2005-2012. [file 12940_2021_751_MOESM3_ESM.docx]

**Additional file 3: Table S2.** Association Between Occupational Exposure to PAHs and Prostate Cancer Risk, Restricting Controls to Men Screened for Prostate Cancer in the Two Years Prior to Interview, PROtEuS, Montreal, Canada, 2005-2012.

|  |  | Prostate cancer | | |
| --- | --- | --- | --- | --- |
| Exposure | n Controls | n Cases | OR**^a^** | 95%CI |
| *PAHs from any source* |  |  |  |  |
| Never exposed | 804 | 1009 | 1.00 | Ref |
| Ever exposed | 441 | 560 | 0.97 | (0.81, 1.16) |
|  |  |  |  |  |
| Duration (per 5-year increment) | 804 | 1009 | 0.99 | (0.96, 1.03) |
|  |  |  |  |  |
| Cumulative exposure |  |  |  |  |
| 0.01 - 275.63 | 228 | 287 | 0.94 | (0.76, 1.17) |
| 275.64 - 1071.64 | 99 | 141 | 1.06 | (0.79, 1.45) |
| > 1071.64 | 114 | 132 | 0.93 | (0.68, 1.26) |
| p for trend |  |  | 0.74 |  |
|  |  |  |  |  |
| *Benzo(a)pyrene* |  |  |  |  |
| Ever exposed | 144 | 182 | 1.02 | (0.77, 1.36) |
|  |  |  |  |  |
| Duration (per 5-year increment) | 144 | 182 | 0.97 | (0.92, 1.02) |
|  |  |  |  |  |
| Cumulative exposure |  |  |  |  |
| 0.01 - 226.95 | 69 | 103 | 1.18 | (0.83, 1.69) |
| 226.96 - 618.95 | 37 | 43 | 0.94 | (0.58, 1.53) |
| > 618.95 | 38 | 36 | 0.77 | (0.45, 1.32) |
| p for trend |  |  | 0.27 |  |
|  |  |  |  |  |
| *PAHs from petroleum* |  |  |  |  |
| Ever exposed | 395 | 514 | 0.99 | (0.82, 1.19) |
|  |  |  |  |  |
| Duration (per 5-year increment) | 395 | 514 | 1.01 | (0.97, 1.04) |
|  |  |  |  |  |
| Cumulative exposure |  |  |  |  |
| 0.01 - 240.50 | 201 | 278 | 1.03 | (0.82, 1.28) |
| 240.51 - 902.38 | 91 | 115 | 0.86 | (0.63, 1.19) |
| > 902.38 | 103 | 121 | 1.01 | (0.74, 1.38) |
| p for trend |  |  | 0.94 |  |
|  |  |  |  |  |
| *PAHs from wood* |  |  |  |  |
| Ever exposed | 34 | 50 | 1.12 | (0.66, 1.89) |
|  |  |  |  |  |
| Duration (per 5-year increment) | 34 | 50 | 1.02 | (0.91, 1.14) |
|  |  |  |  |  |
| Cumulative exposure |  |  |  |  |
| 0.01 - 584.10 | 16 | 25 | 1.14 | (0.57, 2.26) |
| 584.11 - 1608.75 | 8 | 6 | 0.65 | (0.19, 2.16) |
| > 1608.75 | 10 | 19 | 1.48 | (0.56, 3.89) |
| p for trend |  |  | 0.67 |  |
|  |  |  |  |  |
| *PAHs from coal* |  |  |  |  |
| Ever exposed | 46 | 57 | 0.93 | (0.60, 1.44) |
|  |  |  |  |  |
| Duration (per 5-year increment) | 46 | 57 | 0.93 | (0.85, 1.02) |
|  |  |  |  |  |
| Cumulative exposure |  |  |  |  |
| 0.01 - 141.29 | 23 | 36 | 1.12 | (0.63, 1.98) |
| 141.30 - 570.00 | 11 | 14 | 0.98 | (0.42, 2.29) |
| > 570.00 | 12 | 7 | 0.49 | (0.18, 1.32) |
| p for trend |  |  | 0.16 |  |
|  |  |  |  |  |
| *PAHs from other sources* | |  |  |  |
| Ever exposed | 71 | 91 | 0.88 | (0.60, 1.28) |
|  |  |  |  |  |
| Duration (per 5-year increment) | 71 | 91 | 0.99 | (0.92, 1.06) |
|  |  |  |  |  |
| Cumulative exposure |  |  |  |  |
| 0.01 - 300.00 | 37 | 50 | 0.98 | (0.61, 1.57) |
| 300.01 - 825.00 | 16 | 13 | 0.53 | (0.24, 1.15) |
| > 825.00 | 18 | 28 | 0.98 | (0.47, 2.04) |
| p for trend |  |  | 0.81 |  |

Abbreviations: OR, odds ratio; PAHs, polycyclic aromatic hydrocarbons, CI, confidence interval

a-OR adjusted for age, ancestry, education, alcohol drinking, cigarette smoking, body mass index, consumption of fried or grilled food, occupational exposure to benzene, farming and workplace environmental tobacco smoke.
